# S100A8 and S100A9, both transcriptionally regulated by PU.1, promote epithelial-mesenchymal transformation (EMT) and invasive growth of dermal keratinocytes during scar formation post burn

**DOI:** 10.18632/aging.203112

**Published:** 2021-06-07

**Authors:** Zhigang Xu, Chuantao Cheng, Ranran Kong, Yale Liu, Shuang Wang, Yuefeng Ma, Xin Xing

**Affiliations:** 1 Department of Dermatology, The Second Affiliated Hospital of Xi’an Jiaotong University, Xi’an 710000, China; 2 Department of Thoracic Surgery, The Second Affiliated Hospital of Xi’an Jiaotong University, Xi’an 710000, China; 3 Department of Cadre Health, The Second Affiliated Hospital of Xi’an Jiaotong University, Xi’an 710000, China

**Keywords:** burn, keratinocytes, epithelial-mesenchymal transformation, S100A8/A9, PU.1

## Abstract

S100 calcium-binding protein A8 (S100A8) and S100A9 are small molecular weight calcium-binding regulatory proteins that have been involved in multiple chronic inflammatory diseases. However, the role of S100A8 and S100A9 in keratinocytes in wounded skin and how they are regulated during this process are still unclear. Here, we found that S100A8 and S100A9 were both upregulated in burn-wounded skins *in vivo* and thermal-stimulated epidermal keratinocytes *in vitro*, accompanied by increased levels of epithelial-mesenchymal transition (EMT). Then, we demonstrated that upregulation of S100A8 and S100A9 alone or together enhanced characteristics of EMT in normal keratinocytes, manifested by excessive proliferation rate, abnormal ability of cell invasion, and high expression levels of EMT marker proteins. The transcription factor PU box-binding protein (PU.1) bound to the promoter regions and transcriptionally promoted the expression of S100A8 and S100A9 both in the human and mice, and it had strong positive correlations with both S100A8 and S100A9 protein levels in burned skin *in vivo*. Moreover, PU.1 positively regulated expression of S100A8 and S100A9 in a dose-dependent manner, and enhanced EMT of keratinocytes *in vitro*. Finally, through the burn mouse model, we found that PU.1^-/-^ mice displayed a lower ability of scar formation, manifested by smaller scar volume, thickness, and collagen content, which could be enhanced by S100A8 and S100A9. In conclusion, PU.1 transcriptionally promotes expression of S100A8 and S100A9, thus positively regulating epithelial-mesenchymal transformation (EMT) and invasive growth of dermal keratinocytes during scar formation post burn.

## INTRODUCTION

Scar formation is a frequently occurring problem in plastic surgery, which is a skin fibroplasia disorder characterized by abnormal proliferation of dermal and epidermal cell components and excessive deposition of extracellular matrix (ECM) [1]. Scar lesions are often accompanied by itching and tingling symptoms, which not only hinder the beauty and limit the normal function of limbs, but also seriously affect the physical and mental health of patients. It is generally believed that cell types involved in the pathological process of scar formation mainly include dermal fibroblasts and epidermal keratinocytes. Previous studies on the pathological mechanism of scar lesions mainly focused on functions of dermal fibroblasts and their regulation. However, in recent years, more and more studies have revealed that epidermal keratinocytes also play an important role in wound healing and scar formation [2, 3]. During scar formation, keratinocytes gradually lose their characteristics of epithelial cells and acquire features of mesenchymal cells, manifested by decreased ability of cell adhesion and increased ability of migration [4]. However, it is still largely unknown that how is the epithelial-mesenchymal transition (EMT) process regulated in dermal keratinocytes.

S100 calcium-binding protein A8 (S100A8) and S100A9 are small molecular weight calcium-binding regulatory proteins, belonging to the S100 protein family. S100A8 and S100A9, mainly expressed in myeloid cells, including neutrophils, monocytes, and macrophages, and some other cell types with secretory functions, including tissue epithelial cells, endothelial cells, and keratinocytes, have been involved in multiple chronic inflammatory diseases, such as cancer, thrombosis and arthritis [5–9]. Several recent studies revealed that S100A8 and S100A9 are also expressed in fibroblasts, keratinocytes, and other cell components of the skin. S100A8 and S100A9 were robustly upregulated in skin tissues under pathological conditions, such as psoriasis, atopic dermatitis, and wounds, and they contributed to development of these pathological processes [10–12]. In wounded skins, S100A8 and S100A9 expression were induced to promote fibroblast activation and dermal fibrosis. Hence, it is likely that S100A8 and S100A9 play a promoting role in scar formation. However, the role of S100A8 and S100A9 in keratinocytes in wounded skins and their regulation are still unclear.

In a previous study, using proteomics and microarray techniques, we identified that S100A8/A9 were robustly upregulated in the keratinocytes derived from wounded skin tissues. Our elementary study revealed that S100A8/A9 overexpression increased, while silencing S100A8/A9 decreased, the migration capacity of human keratinocytes, accompanied by upregulation of several EMT marker proteins, hinting their potential promoting effect on EMT and invasive growth. In this study, we investigated their exact role in regulating EMT of keratinocytes and scar formation *in vitro* and *in vivo*. Moreover, we explored how S100A8/A9 expression was regulated during this process.

## RESULTS

### S100A8 and S100A9 were both significantly upregulated in post burn skin and heat-stimulated keratinocytes

**Figure 1:**
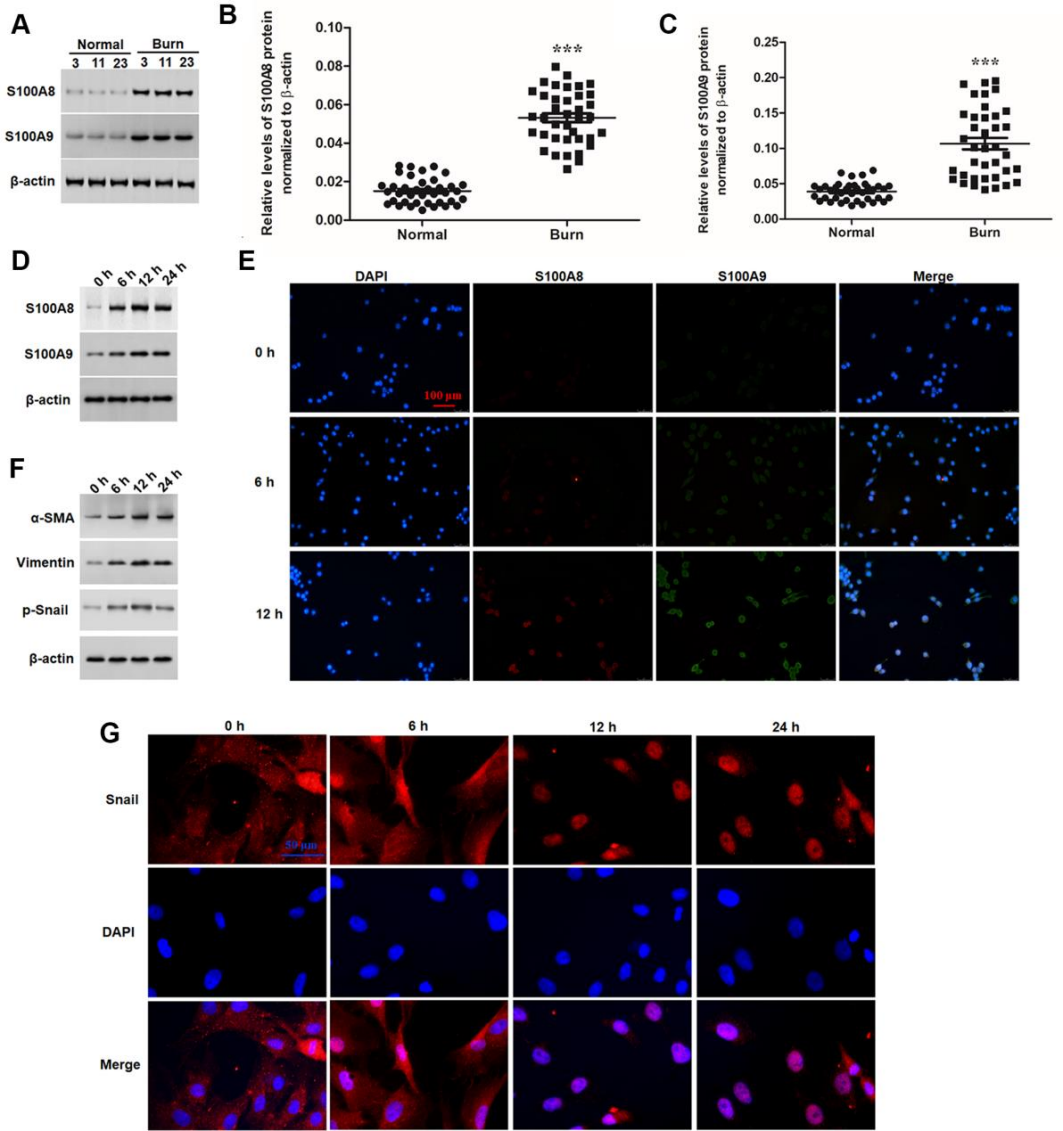
**S100A8 and S100A9 were upregulated in post burn skin and thermal-stimulated keratinocytes.** (**A**) Representative blots for S100A8 and S100A9 in burned and matched normal skin tissues from burn patients. Comparison of (**B**) S100A8 and (**C**) S100A9 in burned and matched normal skin tissues of 38 patients. (**D**) Expression of S100A8 and S100A9 and (**F**) EMT marker proteins in thermal-stimulated keratinocytes was detected with Western blotting at different time points. (**E**) Double immunofluorescence assay for cellular localization of S100A8 and S100A9 and (**G**) immunofluorescence assay for nuclear translocation of Snail protein in thermal-stimulated keratinocytes at different time points. ***P < 0.001.

Firstly, expression of S100A8 and S100A9 proteins was detected in burned and matched normal skin tissues. The results showed S100A8 and S100A9 were both significantly upregulated in post burn skin (Figure 1A–1C). Then, epidermal keratinocytes were isolated from normal human skin, the purity of which were confirmed by FACS assay based on the dual luciferase labels of keratinocyte surface markers CK-14 and p63 (Supplementary Figure 1). Heat-stimulation was applied to treat the keratinocytes *in vitro* for simulating a state of burn injury. Western blotting and double immunofluorescence analyses showed that S100A8 and S100A9 were also significantly upregulated in response to heat stimulation in a time-dependent manner (Figure 1D, 1E). EMT and invasive growth have been recognized as the cellular pathological basis of scar formation after burn. Here, we found that the expression of EMT marker proteins α-SMA, vimentin and p-Snail were significantly induced by heat stimulation, which displayed a synchronous change with S100A8/A9 proteins (Figure 1F). Moreover, as a key regulator of EMT, the nuclear translocation of Snail protein was also induced synchronously (Figure 1G). These data above suggested that S100A8/A9 may be associated with EMT of keratinocytes post heat stimulation.

### S100A8 and S100A9 promote EMT of keratinocytes

**Figure 2:**
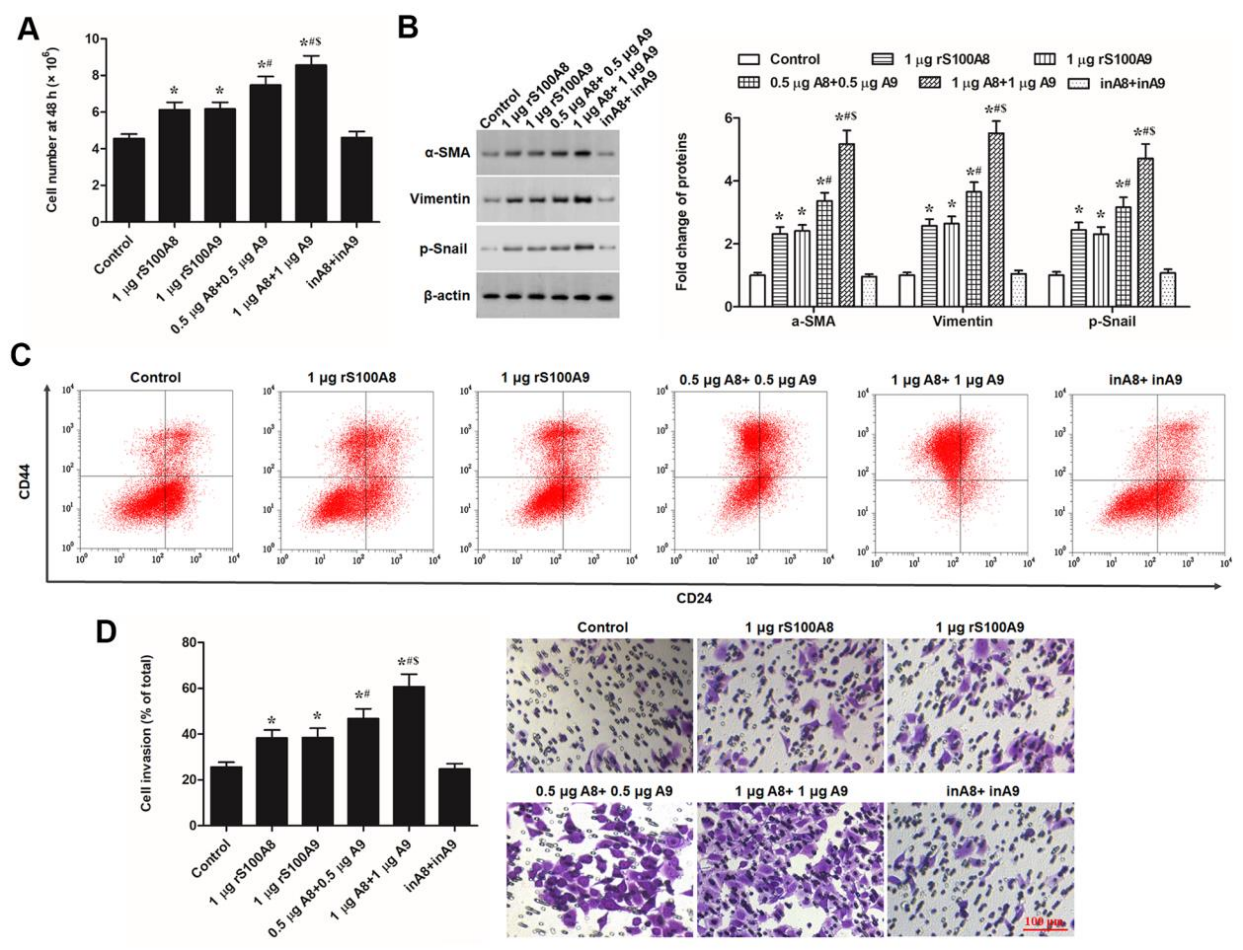
**S100A8 and S100A9 promote cell proliferation, expression of EMT marker proteins, and cell invasion in primary keratinocytes.** Human primary keratinocytes were treated with 1 μg of rS100A8, 1 μg of rS100A9, 0.5 μg of rS100A8 plus 0.5 μg of rS100A9, or 1 μg of rS100A8 plus 1 μg of rS100A9, heat-inactivated rS100A8/rS100A9 were applied as a negative control. After incubation for 48 h, (**A**) cell number, (**B**) expression levels of EMT marker proteins, (**C**) proportion of CD44+CD24- cells, and (**D**) cell invasion, were respectively detected with automatic cell counter, Western blotting, FACS, and Transwell migration assay. *P < 0.05 vs. control, ^#^*P* < 0.05 vs 1 μg rS100A8, ^$^*P* < 0.05 vs. 0.5 μg rS100A8 + 0.5 μg rS100A9.

S100A8 and S100A9 had a positive effect on cell migration and caused a fibroblast-like phenotype in keratinocytes. These signs suggested that that S100A8 and S100A9 caused a transformation from the epithelial phenotype to mesenchymal phenotype in keratinocytes. In this study, we investigated whether S100A8 and S100A9 were involved in EMT keratinocytes. Human recombinant S100A8 and S100A9 proteins (rS100A8 and rS100A9) were incubated with epidermal keratinocytes *in vitro*, and heat-inactivated rS100A8 and rS100A9 were used as the negative control to rule out endotoxin effects. We found that treatment with 1 μg of rS100A8 or 1 μg of rS100A9 alone moderately stimulated cell proliferation (Figure 1A) and invasion (Figure 1D), and expression of EMT marker proteins including α-SMA, vimentin and p-Snail (Figure 1B), while treatment with 0.5 μg of rS100A8 plus 0.5 μg of rS100A9 had a greater promoting effect on cell activity and expression of EMT marker proteins than those of treatment with 1 μg of each recombinant protein alone (Figure 1A, 1B, 1D). Moreover, treatment with 1 μg of rS100A8 plus 1 μg of rS100A9 had a distinctly promoting effect on cell activity and expression of EMT marker proteins, but heat-inactivated rS100A8/A9 had no effect on cell phenotypes and protein expression (Figure 1A, 1B, 1D). To further verify the effect of rS100A8/A9 on EMT of keratinocytes, FACS approach was used to calculate the proportion of CD44^+^CD24^-^ cells in response to treatment. The results showed that the proportion of CD44^+^CD24^-^ cells was increased by the treatment of either rS100A8 or rS100A9 and further increased by co-treatment of rS100A8 plus rS100A9, which was consistent with the changes of cell invasion and levels of EMT marker proteins (Figure 2C). These data revealed that S100A8 and S100A9 synergistically promote EMT of keratinocytes.

### PU.1 was identified as a transcription factor of both S100A8 and S100A9, which promoted EMT of epidermal keratinocytes

**Figure 3:**
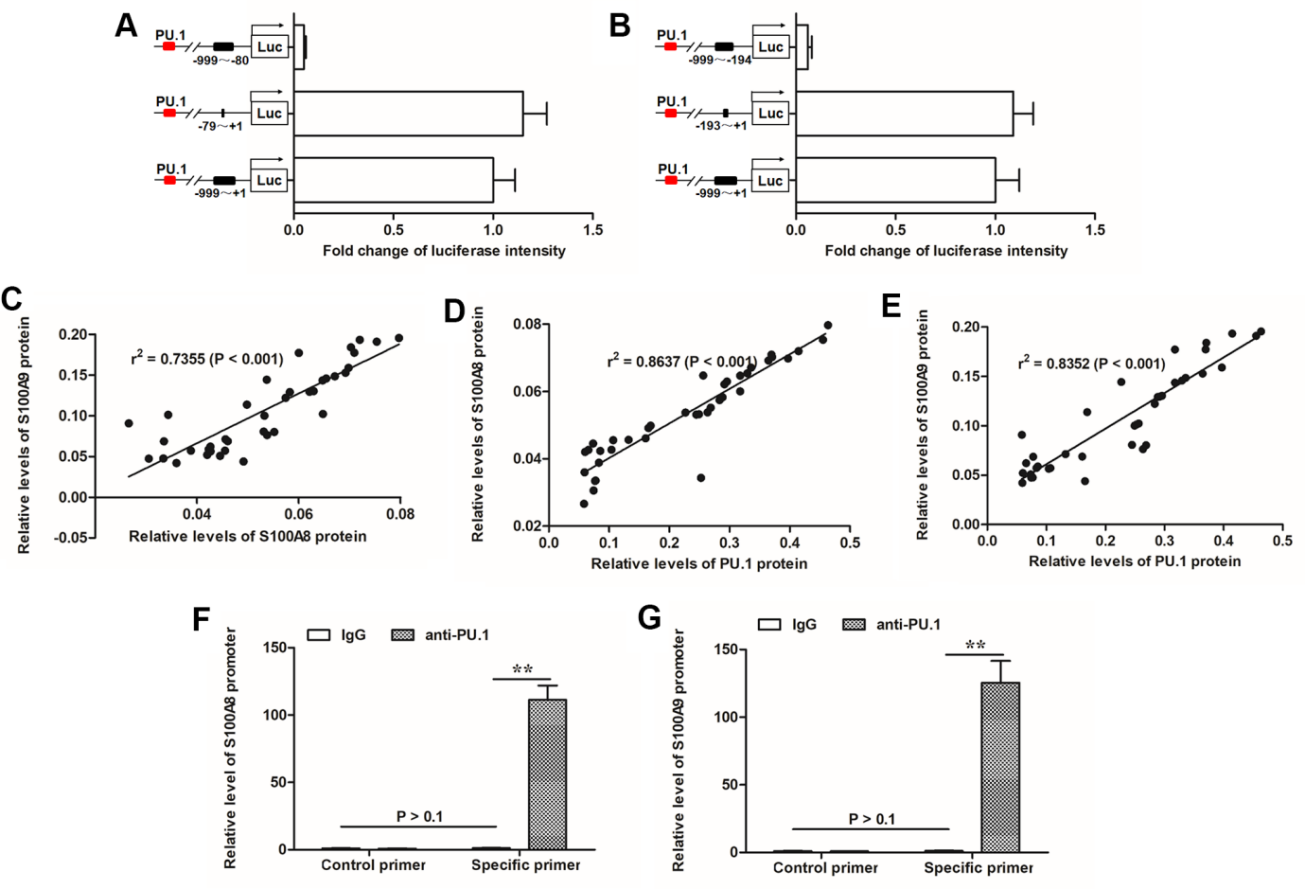
**PU.1 was positive associated with expression of S100A8/9 and identified as a transcription factor of them.** (**A**) Transcriptionally activity of PU.1 on (**A**) S100A8 and (**B**) S100A9 gene promoters was detected by luciferase reporter gene assay. Pearson correlation coefficient assay was used to evaluate the correlations of (**C**) S100A8 and S100A9 expression levels, (**D**) S100A8 and PU.1 expression levels, and (**E**) S100A9 and PU.1 expression levels. ChIP-qPCR assay was used to validate the binding of PU.1 with (**F**) S100A8 promoter and (**G**) S100A9 promoter. **P < 0.01.

S100A8 and S100A9 usually function synergistically, whereas, it is not clear that how S100A8 and S100A9 expressions were regulated. We searched co-expressed regulators with both S100A8 and S100A9 with the online bioinformatics tool ChIPBase. The outputs showed that the transcription factor PU.1 displayed a strong positive correlation with both S100A8 and S100A9 in various human tissues (Supplementary Tables 1, 2). Sequence analyses genes revealed that there was a binding motif in the promoter regions of S100A8 (-79 to -70) and S100A9 (-193 to -182) respectively (Supplementary Figure 2). Luciferase reporter gene assays, based full-length or truncated promoter sequences, verified that PU.1 could activate the transcription of the luciferase reporter genes when the binding motif existed (Figure 3A, 3B). In the burned skin, we found that S100A8 and S100A9 levels displayed a strong positive correlation (Figure 2C). Our results in the burned skin also indicated that PU.1 had a quite strong positive correlation with both S100A8 and S100A9 (Figure 3D, 3E). Chromatin Immunoprecipitation (ChIP) was used to validate the binding of PU.1 with the promoters of S100A8 or S100A9. We observed high abundances of both S100A8 and S100A9 promoter sequences in PU.1-antibody-precipitated DNA mixture (Figure 3F, 3G).

**Figure 4:**
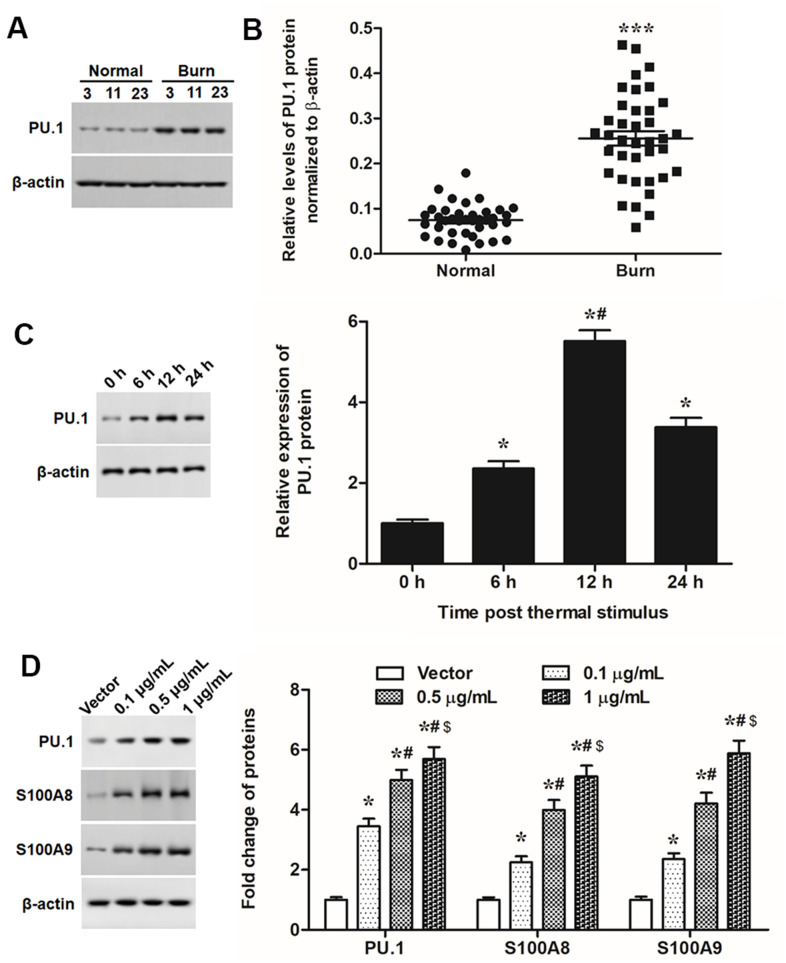
**PU.1 was upregulated in burned skin and promoted expression of S100A8/A9.** (**A**) Representative blots for PU.1 in burned and matched normal skin tissues from burn patients. (**B**) Comparison of PU.1 in burned and matched normal skin tissues of 38 patients. Primary keratinocytes were treated with PU.1 adenoviral expression vector at amounts of 0.1, 0.5 and 1 μg. Empty adenoviral vector was applied as a negative control. After transfection for 48 h, expression levels of (**C**) PU.1 protein and (**D**) EMT marker proteins in thermal-stimulated keratinocytes at different time points. *P < 0.05 vs. 0 h or Vector, ^#^*P* < 0.05 vs. 6 h or 0.5 μg/mL.

**Figure 5:**
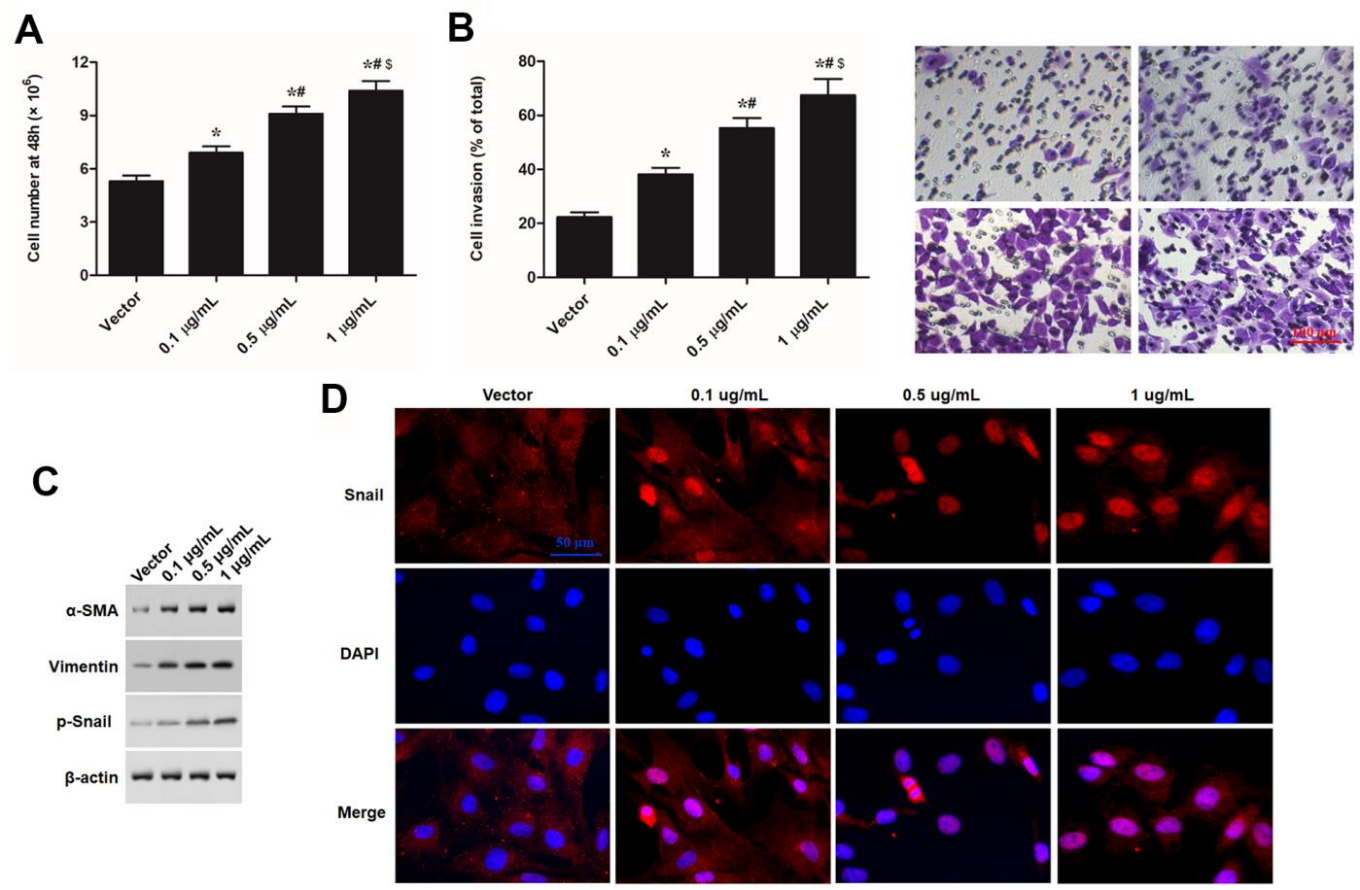
**PU.1 promoted cell proliferation, invasion, and expression of EMT in human epidermal keratinocytes *in vitro*.** Primary keratinocytes were treated with PU.1 adenoviral expression vector at amounts of 0.1, 0.5 and 1 μg. Empty adenoviral vector was applied as a negative control. After transfection for 48 h, (**A**) cell number, (**B**) cell invasion, (**C**) expression levels of EMT marker proteins, and (**D**) nuclear translocation of p-Snail were respectively detected with automatic cell counter, Transwell migration assay Western blotting, and immunofluorescence assay. *P < 0.05 vs. Vector, ^#^*P* < 0.05 vs. 0.1 μg/mL, ^$^*P* < 0.05 vs. 0.5 μg/mL.

Like expression patterns of S100A8 and S100A9, PU.1 was significantly upregulated in burned skin tissues and thermal-stimulated keratinocytes (Figure 4A–4C). Moreover, overexpression of PU.1 promoted expression of S100A8 and S100A9 (Figure 4D), cell proliferation and invasion (Figure 5A, 5B), expression of EMT marker proteins (Figure 5C), as well as the nuclear translocation of Snail (Figure 5D) in a dose-dependent manner. These data demonstrated that PU.1 was a transcription factor of both S100A8 and S100A9, and it played a promoting role in EMT of keratinocytes.

### PU.1^-/-^ displayed lower ability of scar formation, and s100a8 and s100a9 promoted scar development in PU.1^-/-^ burn model mice

**Figure 6:**
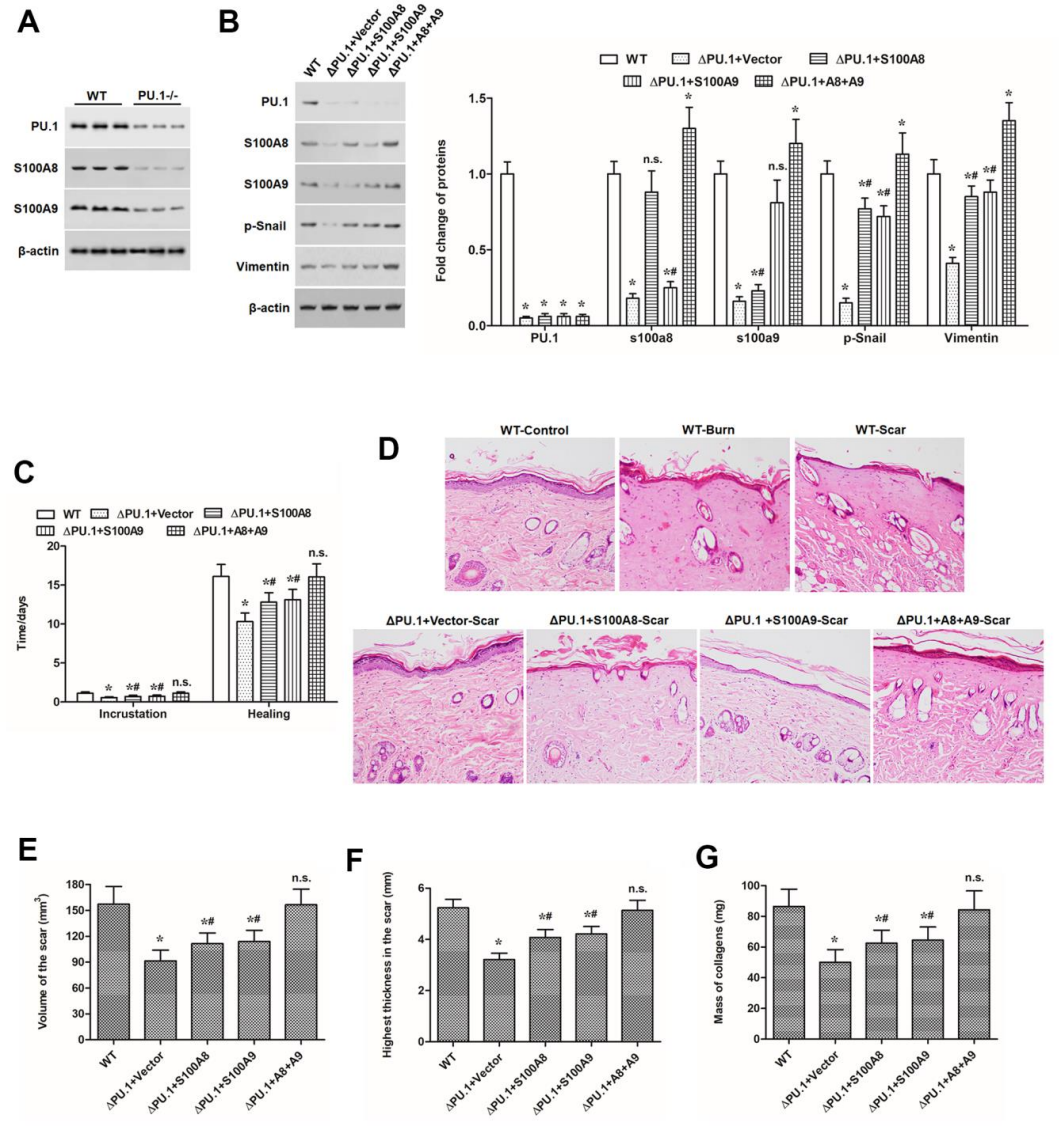
**S100A8 and S100A9 promoted scar development in PU.1^-/-^ burn model mice.** (**A**) expression of PU.1, S100A8 and S100A9 proteins in the skin of WT and skin-specific PU.1 knockout (PU.1^-/-^ or ΔPU.1) mouse. The effect of overexpression of S100A8 or/and S100A9 on (**B**) expression of EMT marker proteins, (**C**) time of wound healing, (**D**) histopathological changes (by HE staining), (**E**) scar volume, (**F**) scar thickness, and (**G**) contents of collagens in PU.1^-/-^ burn model mice. *P < 0.05 vs. WT, ^#^*P* < 0.05 vs. ΔPU.1+Vector.

Finally, we investigated whether the promoting effect of PU.1 on S100A8/9 expression and their mediated keratinocyte EMT conferred its contribution to scar formation *in vivo*. Quite lower expression levels of S100A8 and S100A9 were observed in PU.1^-/-^ mice (Figure 6A). Then, deep second-degree burn model was established in WT and PU.1^-/-^ mice. Our results showed that, compared with the WT mice, PU.1^-/-^ mice displayed much lower levels of S100A8, S100A9 and EMT marker proteins p-Snail and vimentin (Figure 6B) and shorter time of wound healing (Figure 4G), reduced ability of scar development, manifested by smaller scar volume, thickness, and collagen content (Figure 6D–6G). Overexpression of S100A8 or S100A9 alone partially rescued the reduced expression of EMT markers and scar formation ability, and overexpression of both almost rescued those indexes above (Figure 6D–6G).

## DISCUSSION

S100A8 and S100A9 have been famous for their vital catalytic role in chronic inflammatory diseases. Like most studies on regulators in scar formation, which is also regarded as a chronic inflammatory fibrotic disease in the skin, the research focus is also mainly on their role in fibroblast behaviors. In this study, we showed that S100A8 and S100A9 were significantly increased in post-burn skin and thermal-stimulated epidermal keratinocytes, and they promoted EMT of keratinocytes and scar formation. Although there were rare reports on the role of S100A8/A9 in regulating epidermal keratinocyte functions in burn wounded skin, studies on other pathological processes in the epiderma revealed similar functions of S100A8/A9 to the results of our study. For example, in epidermal squamous cell carcinoma, exogenous S100A8/A9 treatment stimulated keratinocyte growth and migration through activated a serious of kinases [13]. Another three studies on different dermatitis, including atopic dermatitis, psoriasis, and epidermodysplasia verruciformis, indicated that S100A8/A9 were upregulated in keratinocytes in response to pathological stimulations and they formed heterodimer to contributed greatly to cell migration and inflammation in dermal keratinocytes [14, 15].

EMT confers to the biological process of the transformation of epithelial cells into mesenchymal phenotype cells, which plays an important role in embryonic development, chronic inflammation, tissue remodeling, cancer metastasis and many kinds of fibrotic diseases. Epithelial cells lose cell polarity and junction with basement membrane, and obtain higher migration and invasion, and extracellular matrix degrading ability through EMT, accompanied by decreased expression of cell adhesion molecules and increased expression of fibrogenic surface molecules and cytoskeletal proteins, such as α-SMA and vimentin [16]. Some studies revealed that EMT of keratinocytes is a key event in scar formation, and inhibition of EMT was recently proposed as a novel therapeutic target for scar development [17–19]. Snail (encoded by SNAI1 gene) is a family of Zinc finger proteins and transcription factors that promote the repression of the adhesion molecule E-cadherin to regulate EMT during embryonic development and many pathological processes [20]. Many members of S100 family, including S100A8 and S100A9, have been involved in EMT in multiple types of cells [21–24]: S100A4 was associated with expression of Snail in human cancers and atrial fibrillation. In this study, we identified S100A8 and S100A9 as positive regulators of Snail, enhancing its phosphorylation and nuclear translocation to promote EMT of epidermal keratinocytes and scar development *in vivo*.

PU-box protein 1 (PU.1) or spleen focus forming virus (SFFV) proviral integration oncogene (SPI1), is an ETS-domain transcription factor and master gene in regulating myeloid and B-lymphoid cell development. A couple of recent studies revealed that PU.1 played an important role in wound healing and tissue fibrosis [25–27]. However, the exact of PU.1 in regulating wound healing and skin fibrosis is still unclear. In this study, we found that PU.1 was significantly upregulated in post burn skin and thermal stimulated dermal keratinocytes, it directly bound to the promoter of both S100A8 and S100A9 genes and promoted their expression, enhancing their mediated EMT of keratinocytes and scar development. There have been two studies reporting the regulatory role of PU.1 on S100A9 expression at the transcriptional level in chronic inflammation processes during aging and progression of promyelocytic leukemia [28, 29], but our data showed that PU.1 not only transcriptionally promoted expression of S100A9 but also S100A8 in humans and mice. In fact, existing studies have suggested that S100A8 and S100A9 not only functioned synergistically but also displayed high synergy in expression.

In conclusion, S100A8 and S100A9 promoted the phosphorylation and nuclear translocation of Snail protein, EMT of epidermal keratinocytes and enhanced scar development. Moreover, they display high synergy in expression, which are transcriptionally regulated by PU.1.

## MATERIALS AND METHODS

### Tissue specimens

A total of 38 tissue specimens were collected from 21 male and 17 female patients (aged 30.1 ± 5.9 years), with second- and third-degree burns admitted within 24 h post burn injury, at the Department of Dermatology, the Second Affiliated Hospital of Xi’an Jiaotong University (Xi’an, China) between Feb, 2017 and May, 2018. Patients with the following exclusion criteria were excluded: severe complications of burns, long-term hormone intake or drug addiction, malignant tumors, and receipt of chemoradiotherapy. During dealing with wounds, burned skin tissues and adjacent normal control skin tissues were respectively obtained. The protocols were approved by the Ethics Committees of the Second Affiliated Hospital of Xi’an Jiaotong University, and all the subjects gave informed consent.

### Culture of primary epidermal keratinocytes and thermal stimulus

Primary epidermal keratinocytes were derived from normal human skin collected from patients who had undergone plastic surgery procedures. The derived tissues were dissected and cut into small pieces, washed with phosphate-buffered saline, and then digested with 0.25% Dispase II (Sigma-Aldrich, Darmstadt, Germany) overnight at 4° C. Then, the suspension was digested with 0.25% trypsin (Sigma-Aldrich) for 30 min. The isolated cells were screened with 70-μm filters to obtain keratinocytes, which were cultured in Dulbecco's modified Eagle's medium (Thermo Fisher Scientific, Waltham, MA, USA) supplemented with 10% fetal bovine serum (FBS; Sigma-Aldrich) at 37° C in an atmosphere with 5% CO_2_. For thermal stimulus, cell cultures were placed into a water heater tank and incubated at 52° C for 30 s, and then cultured in the normal conditions for different time.

### Cell incubation with recombinant proteins, overexpression vectors or siRNAs

Adenoviral expression vector containing human PU.1 (Ad-PU.1), mouse s100a8/a9 (Ad-s100a8 and Ad-s100a9), and their negative control (empty adenoviral plasmid) were purchased from Sangon Biotech (Shanghai, China). Short interfering RNA (siRNAs) were designed and synthesized and validated by RiboBio Co., LTD (Guangzhou, China). Human recombinant S100A8 and S100A9 proteins (rS100A8 and rS100A9) were purchased form Abnova Corporation (Chinese Taipei). Epidermal keratinocytes were subgrown in 6-well plates at a density of 2 × 10^5^ cells/mL. On reaching about 70% confluence, the vectors or siRNAs were transfected into dermal keratinocytes by using the Lipofectamine^®^3000 reagent (Thermo Fisher Scientific). Recombinant proteins were transfected with BioTrek^TM^ (Stratagene, Agilent, Palo Alto, CA, USA).

### Cell proliferation and invasion

The epidermal keratinocytes were seeded at a density of 10^4^ cells/well into 96-well plates. Following incubation, cell number was counted with a Cell Counting Instrument (Countess™ II FL, Thermo Fisher Scientific).

Cell invasion was detected with Transwell Cell assay using Matrigel-coated Transwell chambers (8.0 μm, Corning, Kennebunk, ME, USA), respectively. After 48 h of transfection, 10^5^ cells were plated in the wells above in 200 μL serum-free medium, and 800 μL of medium supplemented with 10% FBS was added to the bottom wells. After incubation at 37° C with 5% CO_2_ for 48 h, the Transwell membrane was fixed with methanol for 20 min, stained with 0.1% crystal violet, and counted the cell numbers of 5 fields (upper and lower, left and right, middle) to obtain the mean value via light microscopy.

### Quantitative polymerase chain reaction (qPCR)

Total RNA from the tissues and keratinocytes was extracted by using the Total RNA Purification Kit containing DNase I (Promega Corporation, Madison, WI, USA), and then applied in cDNA synthesis. RNA abundances were detected using a final volume of 25-μL reaction system containing SYBR ExScript qPCR kit (Takara, Dalian, China) in an IQ^5^ system (Bio-Rad). The reaction system was incubated under the following conditions: 95° C for 3 min, followed by denaturation at 94° C for 15 s, annealing at 55° C for 25 s and extension at 72° C for 15 s for 35 cycles. 18S RNA was used as an internal reference gene control.

### Western blotting

Total Protein Extraction Kit (Promega) was used to extract total protein. Twenty-five micrograms of protein sample were separated by polyacrylamide gel at 130 V for 2 h and then electro-transferred onto the polyvinylidene fluoride membrane (Millipore, Boston, MA, USA). The following primary antibodies (Abcam, Cambridge, UK) were respectively used to incubate with the membrane at 4° C overnight: Rabbit polyclonal antibody to phosphorylated SNAIL (p-Snail S246, 1:500 dilution, ab63568), Rabbit monoclonal antibody [E184] to α-SMA (1:400, ab209435), Rabbit polyclonal antibody to Vimentin (1:600, ab137321), Rabbit monoclonal antibody [EPR22624-20] to PU.1 (1:300, ab227835), Rabbit monoclonal antibody [EPR3554] to MRP8/S100A8 (1:300, ab219370), Rabbit monoclonal antibody [EPR22332-75] to S100A9 (1:300, ab242945), and Rabbit monoclonal antibody [EPR16769] to β-Actin (1:800, ab179467). After incubation with appropriate secondary antibodies (horseradish peroxidase-conjugated Goat-anti-Rabbit IgG) at 37° C for 1 h. After elution, the abundances of the proteins were detected using a ChemiDoc XRS Imaging System (Bio-Rad).

### Prediction and validation of PU.1 binding to the promoter regions of S100A8/S100A9

The co-expression of PU.1 and S100A8/S100A9 was evaluated by the online bioinformatics tool ChIPBase version 2.0 (http://rna.sysu.edu.cn/chipbase/). PU.1 binding motif was also searched on the promoter regions of S100A8/S100A9 using ChIPBase 2.0.

ChIP assay was performed to validate the binding of PU.1 with the promoter sequences of S100A8 or S100A9 using ChIP-IT Expression Chromatin Immunoprecipitation Kits (Active motif, Carlsbad, CA, USA). In brief, cells were fixed with 4% formaldehyde for 10 min. 100 ~500 bp DNA fragments were obtained from lysed cells by ultrasonic crushing. The immuno-complexes were precipitated using PU.1 antibody and normal serum IgG through rotational incubation at 4° C overnight. The same amount of chromatin not incubated with antibodies was used as the input control. Enrichment of promoter sequences of S100A8 or S100A9 was then detected with qPCR in the precipitated DNA.

Luciferase reporter gene assay was used to evaluate the transcription regulatory activity of PU.1 on S100A8/S100A9 genes. Vectors containing different truncated promoter sequences were cloned and inserted into pGL3-basic vector (Promega, Madison, WI, USA) with PU.1 at the upstream. The assay was performed according to the manufacturer’s instructions.

### Establishment of experimental burn model in wild type and PU.1 knockout mouse

Clean-grade wild type (WT) and skin-specific PU.1 knockout (PU.1^-/-^ or ΔPU.1) BALB/c mice (about 20 g) were purchased from Laboratory Animal Center, Shanghai Institutes for Biological Sciences (Shanghai, China). For the generation of ΔPU.1 mouse, in brief, keratin14-CRE transgenic tool mice and PU. 1 conditional knockout (PU.1^loxp/loxp^) mice mated two generations to obtain the mice with PU. 1 gene specific deletion in keratinocytes from embryonic day 14.5. After adaptive feeding for two weeks, deep second-degree experimental burn model was established: mice were intraperitoneally injected with 7% chloral hydrate, and the injection volume was 5 mL/kg. After successful anesthesia, hair on the back of the mice was removed to expose the skin, and then sterilized with 75% ethanol. Then the back of the mice was contacted with a 92° C -copper cylinder with 1.5 cm bottom diameter for 4 s, resulting in burn wound. Adeno-associated-viral vectors delivering S100A8 or/and Ad-S100A9 were locally injected into the epidermis at 24 h before burn. After the burn wound of the mice was healed, the formation of scar on the back of the mice were observed regularly. Until the scar height increases no more, the mice were sacrificed, and the keloids were carefully obtained for measurement of the scar thickness at the lowest and highest sites, the scar volume, and the content of total collagens. And skin tissues (with an area of ~1 cm^2^) were collected for histological examination with HE staining) and expression of related proteins with Western blotting.

### Immunofluorescence assay for the colocalization of S100A8/A9 proteins and translocation of Snail protein

The cell climbing sheets were quenched with 3% hydrogen peroxide for 15 min, blocked with normal goat serum for 30 min to eliminate non-specific binding and then incubated overnight at 4° C with primary immunofluorescence-labelled antibodies against S100A8 (Anti-S100A8/MRP8 Antibody clone CF-145, FITC, Product# ALS12499), S100A9 (Anti-S100A9 Polyclonal Antibody, FITC, Product# PA1-46489, Invitrogen) or Snail (Mouse monoclonal Antibody [CL3700] to SNAIL-N-terminal, ab224731). The next day, the sheets were washed in PBS and incubated in ABC (PK-6100 Elite ABC Kit; Vector Laboratories) for 30 min. Following washing in PBS, DAB solution (SK4100; Vector Laboratories) was used to incubated with the sheets for 5 min, followed by hematoxylin staining. Finally, the sheets were counterstained with hematoxylin and images were taken with a FSX100 microscope (Olympus, Tokyo, JPN) and analyzed by Image-Pro Plus 6.0 system.

### Fluorescence activated cell sorting (FACS)

FACS was used to verify the purity of isolated primary keratinocytes (based on keratinocytes specific surface marker proteins cytokeratin-14/CK-14 and p63) and EMT phenotype of primary keratinocytes treated with different recombinant proteins (based on EMT marker proteins CD44 and CD24). In brief, keratinocytes were tagged with either green or red FITC-antibodies and then analyzed with a Attune™ NxT Flow Cytometer (Invitrogen). The ratio of CK-14^+^p63^+^ cells represent the purity of keratinocytes, and higher percentage of CD44^+^CD24^-^ indicates higher EMT level of keratinocytes.

### Statistical analysis

Each measurement was obtained from at least triple experiments and shown as the mean ± standard error of mean (SEM). Data analysis was performed with SPSS version 23.0. Pearson correlation coefficient assay was used to evaluate the correlation of PU.1-S100A8-S100A9. Statistical significance difference was set at *P* < 0.05 using one-way variation analysis followed by student’s *t*-test.

## Supplementary Material

Supplementary Figures

Supplementary Tables

## References

[1] Chun Q, ZhiYong W, Fei S, XiQiao W. Dynamic biological changes in fibroblasts during hypertrophic scar formation and regression. Int Wound J. 2016; 13:257–62. 10.1111/iwj.12283 24802644 PMC7949937

[2] Edriss AS, Mešták J. Epidermal Keratinocytes May Have an Important role in Hypertrophic Scarring Pathogenesis: an Immunohistochemical Study (Using P63 and Ki-67 Staining). Ann Burns Fire Disasters. 2005; 18:133–39. 21990995 PMC3187988

[3] Li B, Gao C, Diao JS, Wang DL, Chu FF, Li Y, Wang G, Guo SZ, Xia W. Aberrant Notch signalling contributes to hypertrophic scar formation by modulating the phenotype of keratinocytes. Exp Dermatol. 2016; 25:137–42. 10.1111/exd.12897 26566963

[4] Nordin A, Chowdhury SR, Saim AB, Bt Hj Idrus R. Effect of Kelulut Honey on the Cellular Dynamics of TGFβ-Induced Epithelial to Mesenchymal Transition in Primary Human Keratinocytes. Int J Environ Res Public Health. 2020; 17:3229. 10.3390/ijerph17093229 32384749 PMC7246951

[5] Wang S, Song R, Wang Z, Jing Z, Wang S, Ma J. S100A8/A9 in Inflammation. Front Immunol. 2018; 9:1298. 10.3389/fimmu.2018.01298 29942307 PMC6004386

[6] Frohberger SJ, Fercoq F, Neumann AL, Surendar J, Stamminger W, Ehrens A, Karunakaran I, Remion E, Vogl T, Hoerauf A, Martin C, Hübner MP. S100A8/S100A9 deficiency increases neutrophil activation and protective immune responses against invading infective L3 larvae of the filarial nematode Litomosoides sigmodontis. PLoS Negl Trop Dis. 2020; 14:e0008119. 10.1371/journal.pntd.0008119 32107497 PMC7064255

[7] Schneider RK, Schenone M, Ferreira MV, Kramann R, Joyce CE, Hartigan C, Beier F, Brümmendorf TH, Germing U, Platzbecker U, Büsche G, Knüchel R, Chen MC, et al. Rps14 haploinsufficiency causes a block in erythroid differentiation mediated by S100A8 and S100A9. Nat Med. 2016; 22:288–97. 10.1038/nm.4047 26878232 PMC4870050

[8] Xiao X, Yang C, Qu SL, Shao YD, Zhou CY, Chao R, Huang L, Zhang C. S100 proteins in atherosclerosis. Clin Chim Acta. 2020; 502:293–304. 10.1016/j.cca.2019.11.019 31794767

[9] Huang SJ, Ding ZN, Xiang HX, Fu L, Fei J. Association Between Serum S100A8/S100A9 Heterodimer and Pulmonary Function in Patients with Acute Exacerbation of Chronic Obstructive Pulmonary Disease. Lung. 2020; 198:645–52. 10.1007/s00408-020-00376-9 32661658

[10] Defrêne J, Berrazouane S, Esparza N, Pagé N, Côté MF, Gobeil S, Aoudjit F, Tessier PA. Deletion of S100a8 and S100a9 Enhances Skin Hyperplasia and Promotes the Th17 Response in Imiquimod-Induced Psoriasis. J Immunol. 2021; 206:505–14. 10.4049/jimmunol.2000087 33361205

[11] Kim MJ, Im MA, Lee JS, Mun JY, Kim DH, Gu A, Kim IS. Effect of S100A8 and S100A9 on expressions of cytokine and skin barrier protein in human keratinocytes. Mol Med Rep. 2019; 20:2476–83. 10.3892/mmr.2019.10454 31322196

[12] Schlecht A, Boneva S, Gruber M, Zhang P, Horres R, Bucher F, Auw-Haedrich C, Hansen L, Stahl A, Hilgendorf I, Agostini H, Wieghofer P, Schlunck G, et al. Transcriptomic Characterization of Human Choroidal Neovascular Membranes Identifies Calprotectin as a Novel Biomarker for Patients with Age-Related Macular Degeneration. Am J Pathol. 2020; 190:1632–42. 10.1016/j.ajpath.2020.04.004 32339498

[13] Argyris PP, Slama Z, Malz C, Koutlas IG, Pakzad B, Patel K, Kademani D, Khammanivong A, Herzberg MC. Intracellular calprotectin (S100A8/A9) controls epithelial differentiation and caspase-mediated cleavage of EGFR in head and neck squamous cell carcinoma. Oral Oncol. 2019; 95:1–10. 10.1016/j.oraloncology.2019.05.027 31345374 PMC6662626

[14] D’Amico F, Granata M, Skarmoutsou E, Trovato C, Lovero G, Gangemi P, Longo V, Pettinato M, Mazzarino MC. Biological therapy downregulates the heterodimer S100A8/A9 (calprotectin) expression in psoriatic patients. Inflamm Res. 2018; 67:609–16. 10.1007/s00011-018-1147-6 29605872

[15] Podgórska M, Ołdak M, Marthaler A, Fingerle A, Walch-Rückheim B, Lohse S, Müller CS, Vogt T, Ustav M, Wnorowski A, Malejczyk M, Majewski S, Smola S. Chronic Inflammatory Microenvironment in Epidermodysplasia Verruciformis Skin Lesions: Role of the Synergism Between HPV8 E2 and C/EBPβ to Induce Pro-Inflammatory S100A8/A9 Proteins. Front Microbiol. 2018; 9:392. 10.3389/fmicb.2018.00392 29563902 PMC5845987

[16] Katsuno Y, Qin J, Oses-Prieto J, Wang H, Jackson-Weaver O, Zhang T, Lamouille S, Wu J, Burlingame A, Xu J, Derynck R. Arginine methylation of SMAD7 by PRMT1 in TGF-β-induced epithelial-mesenchymal transition and epithelial stem-cell generation. J Biol Chem. 2018; 293:13059–72. 10.1074/jbc.RA118.002027 29907569 PMC6109915

[17] Stone RC, Pastar I, Ojeh N, Chen V, Liu S, Garzon KI, Tomic-Canic M. Epithelial-mesenchymal transition in tissue repair and fibrosis. Cell Tissue Res. 2016; 365:495–506. 10.1007/s00441-016-2464-0 27461257 PMC5011038

[18] Yuan FL, Sun ZL, Feng Y, Liu SY, Du Y, Yu S, Yang ML, Lv GZ. Epithelial-mesenchymal transition in the formation of hypertrophic scars and keloids. J Cell Physiol. 2019; 234:21662–69. 10.1002/jcp.28830 31106425

[19] Yang S, Zhao J, Huang S, Shu B, Yang R, Chen L, Xu Y, Xie J, Liu X, Jia J, Qi S. Reduced hydration-induced decreased caveolin-1 expression causes epithelial-to-mesenchymal transition. Am J Transl Res. 2020; 12:8067–83. 33437382 PMC7791524

[20] Li CF, Chen JY, Ho YH, Hsu WH, Wu LC, Lan HY, Hsu DS, Tai SK, Chang YC, Yang MH. Snail-induced claudin-11 prompts collective migration for tumour progression. Nat Cell Biol. 2019; 21:251–62. 10.1038/s41556-018-0268-z 30664792

[21] Zhang M, Zheng S, Jing C, Zhang J, Shen H, Xu X, Lin J, Zhang B. S100A11 promotes TGF-β1-induced epithelial-mesenchymal transition through SMAD2/3 signaling pathway in intrahepatic cholangiocarcinoma. Future Oncol. 2018; 14:837–47. 10.2217/fon-2017-0534 29569474

[22] Al-Ismaeel Q, Neal CP, Al-Mahmoodi H, Almutairi Z, Al-Shamarti I, Straatman K, Jaunbocus N, Irvine A, Issa E, Moreman C, Dennison AR, Emre Sayan A, McDearmid J, et al. ZEB1 and IL-6/11-STAT3 signalling cooperate to define invasive potential of pancreatic cancer cells via differential regulation of the expression of S100 proteins. Br J Cancer. 2019; 121:65–75. 10.1038/s41416-019-0483-9 31123345 PMC6738112

[23] Lv H, Hou H, Lei H, Nie C, Chen B, Bie L, Han L, Chen X. MicroRNA-6884-5p Regulates the Proliferation, Invasion, and EMT of Gastric Cancer Cells by Directly Targeting S100A16. Oncol Res. 2020; 28:225–36. 10.3727/096504019X15753718797664 31796150 PMC7851531

[24] Tian X, Cao Z, Ding Q, Li Z, Zhang C. Prognostic value of multiple epithelial mesenchymal transition-associated proteins in intrahepatic cholangiocarcinoma. Oncol Lett. 2019; 18:2059–65. 10.3892/ol.2019.10522 31423278 PMC6614681

[25] Cooper L, Johnson C, Burslem F, Martin P. Wound healing and inflammation genes revealed by array analysis of ‘macrophageless’ PU.1 null mice. Genome Biol. 2005; 6:R5. 10.1186/gb-2004-6-1-r5 15642097 PMC549066

[26] Liu Q, Zhang Y, Yang S, Wu Y, Wang J, Yu W, Liu Y. PU.1-deficient mice are resistant to thioacetamide-induced hepatic fibrosis: PU.1 finely regulates Sirt1 expression via transcriptional promotion of miR-34a and miR-29c in hepatic stellate cells. Biosci Rep. 2017; 37:BSR20170926. 10.1042/BSR20170926 29162670 PMC5725609

[27] Wohlfahrt T, Rauber S, Uebe S, Luber M, Soare A, Ekici A, Weber S, Matei AE, Chen CW, Maier C, Karouzakis E, Kiener HP, Pachera E, et al. PU.1 controls fibroblast polarization and tissue fibrosis. Nature. 2019; 566:344–49. 10.1038/s41586-019-0896-x 30700907 PMC6526281

[28] Swindell WR, Johnston A, Xing X, Little A, Robichaud P, Voorhees JJ, Fisher G, Gudjonsson JE. Robust shifts in S100a9 expression with aging: a novel mechanism for chronic inflammation. Sci Rep. 2013; 3:1215. 10.1038/srep01215 23386971 PMC3564041

[29] Zhu Y, Zhang F, Zhang S, Deng W, Fan H, Wang H, Zhang J. Regulatory mechanism and functional analysis of S100A9 in acute promyelocytic leukemia cells. Front Med. 2017; 11:87–96. 10.1007/s11684-016-0469-4 28063140

