# Supplementary material for: S100A8 and S100A9, both transcriptionally regulated by PU.1, promote epithelial-mesenchymal transformation (EMT) and invasive growth of dermal keratinocytes during scar formation post burn
Source: Aging (Albany NY). 2021 Jun 7;13(11):15523–37. doi: 10.18632/aging.203112 (PMC8221299; doi:10.18632/aging.203112)
Supplement: Supplementary Figures [file aging-13-203112-s001.pdf]

SUPPLEMENTARY FIGURES

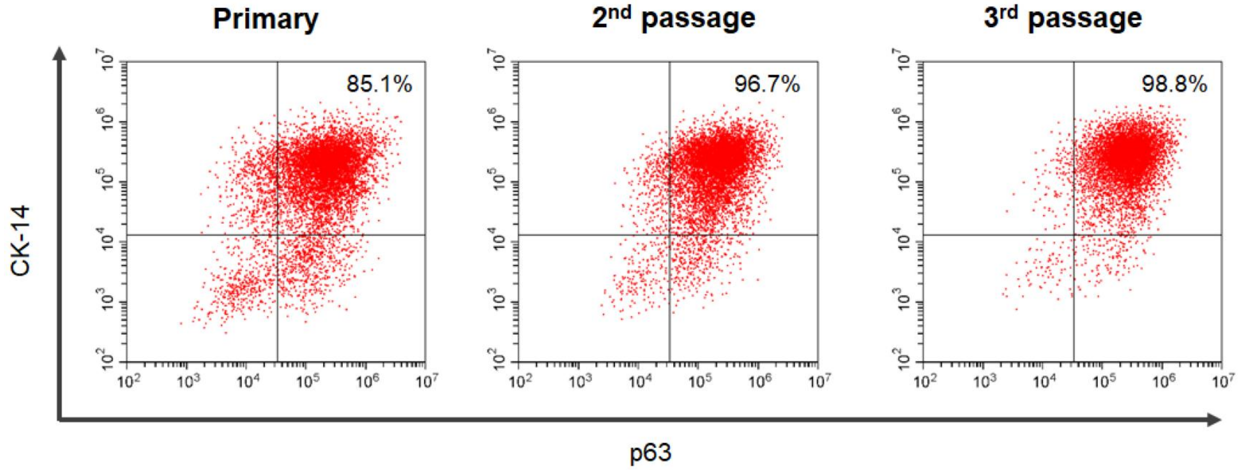

Supplementary Figure 1. Dual fluorescent antibodies, specific surface marker proteins cytokeratin-14 (CK-14) and p63, were used to label the keratinocytes, and FACS was used to verify the purity of isolated primary keratinocytes, the 2nd passage, and the 3rd passage.

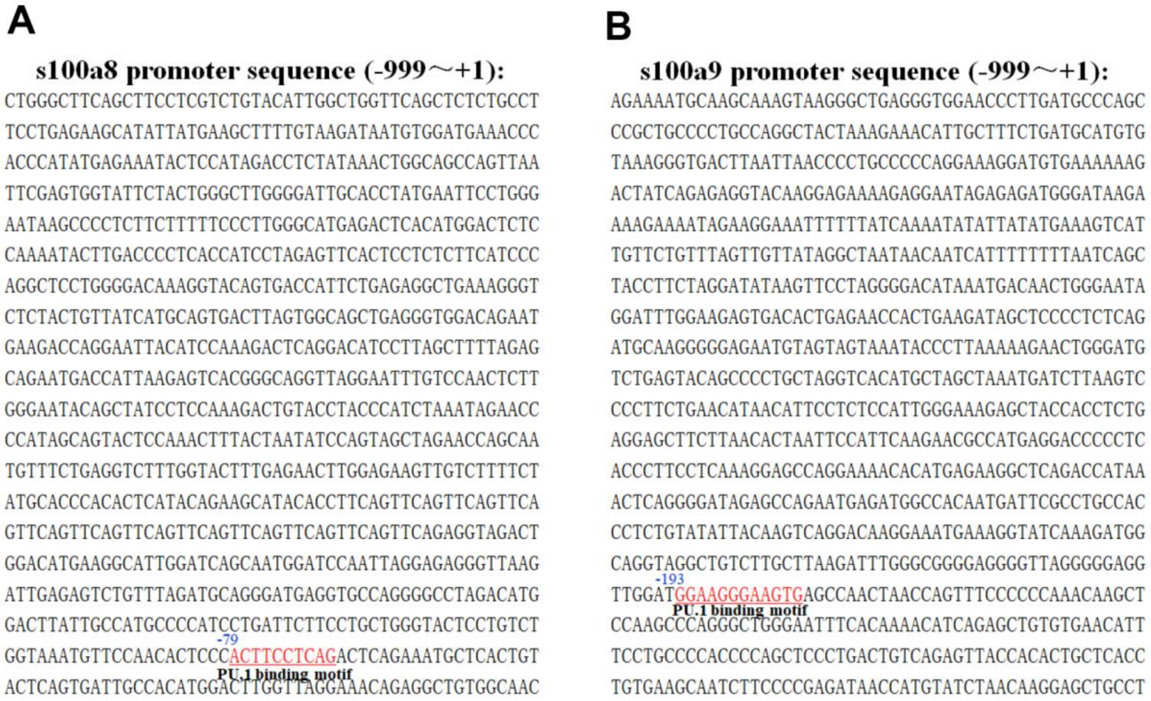

Supplementary Figure 2. Sequence analysis for the promoter region of s100a8 and s100a9 genes. (**A**) The position of PU.1 binding motif at s100a8 promoter region; (**B**) The position of PU.1 binding motif at s100a9 promoter region.
