# Supplementary material for: S100A8 and S100A9, both transcriptionally regulated by PU.1, promote epithelial-mesenchymal transformation (EMT) and invasive growth of dermal keratinocytes during scar formation post burn
Source: Aging (Albany NY). 2021 Jun 7;13(11):15523–37. doi: 10.18632/aging.203112 (PMC8221299; doi:10.18632/aging.203112)
Supplement: Supplementary Tables [file aging-13-203112-s002.pdf]

SUPPLEMENTARY TABLES

Supplementary Table 1. Overview of co-expression patterns (log_2-scale) between SPI1 and S100S8 in 10 tissues.

Tissues	Sample number	Pearson coefficient r	*p*-value (*t*-test, 2 tail)
Blood	444	0.9261	3.6E-189
Skin mixed tissue	813	0.685	1.14E-113
Brain mixed tissue	1146	0.5211	8.59E-81
Blood vessel mixed tissue	604	0.6012	1.26E-60
Heart mixed tissue	376	0.6809	1.52E-52
Stomach tissue	173	0.7796	1.39E-36
Adipose mixed tissue	515	0.4698	1.22E-29
Muscle tissue	396	0.474	1.41E-23
Spleen tissue	99	0.772	8.49E-21
Colon tissue	304	0.4856	2.18E-19

Supplementary Table 2. Overview of co-expression patterns (log_2-scale) between SPI1 and S100S9 in 10 tissues.

Tissues	Sample number	Pearson coefficient r	*p*-value (*t*-test, 2 tail)
Blood	444	0.9505	1.8E-226
Skin mixed tissue	813	0.7012	2.7E-121
Brain mixed tissue	1146	0.5261	1.48E-82
Blood vessel mixed tissue	604	0.7186	5.1E-97
Heart mixed tissue	376	0.7644	2.82E-73
Stomach tissue	173	0.8133	4.46E-42
Adipose mixed tissue	515	0.5597	8.64E-44
Muscle tissue	396	0.5243	2.44E-29
Spleen tissue	99	0.8142	1.23E-24
Liver tissue	110	0.7945	3.87E-25
